# Advanced glycation end products and diabetes and other metabolic indicators

**DOI:** 10.1186/s13098-022-00873-2

**Published:** 2022-07-25

**Authors:** Tian Jiang, Yi Zhang, Fang Dai, Chao Liu, Honglin Hu, Qiu Zhang

**Affiliations:** 1 Department of Endocrinology, The First Affiliated Hospital of Anhui Medical University, Hefei, 230022 Anhui China; 2 Department of Maternal, Child and Adolescent Health, School of Public Health, Anhui Medical University, No 81 Meishan Road, Hefei, 230032 Anhui China

**Keywords:** Advanced glycation end products, Diabetes mellitus, Metabolic indicators, BMI, TG, TC

## Abstract

**Background:**

Diabetes is a global concern among adults. Previous studies have suggested an association between different screening methods and diabetes; however, increasing evidence has suggested the importance of early screening for diabetes mellitus (DM) and its influencing factors. In this study, we aimed to explore whether the non-invasive detection of advanced glycation end products (AGEs) in the early screening of DM in the Chinese community and whether body mass index (BMI) and metabolic indexes could moderate this relationship.

**Methods:**

Three community health service centers in Hefei that signed the medical consortium agreement with the First Affiliated Hospital of Anhui Medical University were selected to screen the population aged 30–90 years in each community using a multi-stage cluster sampling method from January 2018 to January 2019. Univariate analysis of variance was used to compare the differences in general data, biochemical indexes, skin AGEs levels, and blood glucose among groups. In addition, a multivariable logistic regression analysis was performed.

**Results:**

A total of 912 patients with a community health physical examination and no history of diabetes were selected, excluding those with missing values > 5%. Finally, 906 samples were included in the study with an effective rate of 99.3%. The prevalence in the normal, impaired glucose tolerance, and DM groups were 79.8%, 10.0%, and 10.2%, respectively. By dividing AGE by quartile, AGE accumulation was classified as ≤ P25, P25–P50, P50–P75, and > P75. Higher AGE accumulation (*χ*^2^ = 37.95), BMI (*χ*^2^ = 12.20), systolic blood pressure (SBP) (*χ*^2^ = 8.46), triglyceride (TG) (*χ*^2^ = 6.23), and older age (*χ*^2^ = 20.11) were more likely to have a higher prevalence of fasting blood glucose (FBG). The analyses revealed significant correlations between AGE accumulation, BMI, TG, total cholesterol (TC), and FBG (*P* < 0.05).

**Conclusion:**

As the findings indicate, priority should be given to the quality of metabolic-related indicators, such as BMI, TG, and TC, employed to effectively reduce the FBG of Chinese participants with high AGE accumulation. Skin autofluorescence may prove to be a rapid and non-invasive method for assessing the metabolic progression of all glucose level layers.

## Background

The prevention of non-communicable diseases (NCDs) are an important task in the fields of public health and clinical medicine. NCDs, including diabetes and obesity, have become an important factor affecting human survival rate and mortality [1]. According to statistics, 1 in 10 adults live with diabetes worldwide, and almost half are undiagnosed [2]. This number may increase the threat of a pandemic with type 2 diabetes mellitus (T2DM), and relative data also predict that the prevalence of T2DM will reach 783 million people worldwide in 2045 [3]. Approximately 422 million people worldwide currently live with diabetes, most of whom live in low- and middle-income countries and 1.5 million people die from diabetes directly each year [4]. The number of cases and the prevalence of diabetes have been steadily increasing over the past few decades. Another phenomenon, prediabetes, also known as impaired glucose regulation (IGT), is the metabolic state between normal glucose tolerance and diabetes. In a previous nationally representative cross-sectional survey conducted in Mainland China in 2018–2019, a total of 173,642 people participated, and the estimated prevalence of diabetes and prediabetes was 12.4% and 38.1%, respectively [1], which is higher than that in 2013 [5]. Since the epidemic rate of diabetes is rising annually [6], early diagnosis and intervention for prediabetes are of great significance.

Ceriello et al. raised that "metabolic memory" plays an important role in the development of long-term metabolic complications in patients with type 1 and type 2 diabetes [7]. Metabolic memory is to a method that can account for significant differences in the incidence and severity of complications between blood glucose and diabetes [8]. How to better determine the index of "metabolic memory" is particularly important. In other major metabolic pathways involved in the pathogenesis of advanced diabetes complications [e.g., oxidative stress, inflammation, and advanced glycation end products (AGEs)], the measurement of AGEs seems to be one of the most appropriate methods to assess the true impact of chronic hyperglycemia [9], and research evidence suggests that there is an association between "metabolic memory" and AGEs [7]. Studies have shown that in T2DM, the accumulation of AGEs in tissues increases, and circulating AGEs may be a predictor of future diabetes risk [10]. Moreover, many risk factors are known, but not all factors taken together fully explain the risk of diabetic complications, suggesting that other pathophysiological mechanisms are at work.

An increase in tissue AGEs may be a surrogate mechanism. As a non-invasive clinical tool, skin autofluorescence (SAF) can be used to assess the accumulation of AGEs [11–13]. These studies suggest that glycemic control has a long-term impact on the development and progression of diabetic complications and that all eligible studies showed a positive association between SAF and one or more diabetic complications (all-cause mortality, cardiovascular mortality, microvascular and macrovascular complications, neuropathy, and kidney disease) [14]. Therefore, it is necessary to detect AGE levels effectively and quickly. Zhu et al. developed a non-invasive diabetes detector (DM Scan) by applying the optical detection technology of AGEs and confirmed that AGEs on the skin, as a screening indicator for diabetes, have the advantages of being rapid, non-invasive, consumable, and have no risk of cross-infection [15]. In addition, increased AGE levels have been associated with many microvascular diabetic complications [16], such as lipid profile [17] and obesity, further explaining why we explored the association between metabolic index and AGE in newly diagnosed patients with diabetes and prediabetes. Melin et al. demonstrated that there is an association between receptors for AGEs and lipid-lowering drugs [17].

To some extent, these studies on AGEs can promote the formation and development of diabetes and prediabetes and provide indirect evidence, while AGEs and the establishment of AGE-diabetes and prediabetes require a comprehensive model, and the phenomenon of diabetes and prediabetes should be considered comprehensively. Currently, a large sample of invasive serological examinations is difficult to popularize in some grassroots communities, and there is still a lack of relevant reports on whether non-invasive skin detection of AGEs is suitable for the early risk screening of diabetes. To address this important issue, we performed a large-scale, multicenter, cross-sectional study on the different communities in three communities in China. This study aimed to explore the interaction association between AGEs and diabetes and prediabetes. Further, in such a case of exposure to the AGEs, participants whose other indexes combined with AGEs were associated with diabetes and prediabetes. Therefore, this study aimed to explore the association between body mass index (BMI), AGE, lipid-related indicators, and blood glucose to consider the influence of exposure to multiple risk factors on blood glucose and its association with prediabetes. In addition, the author aimed to explore the application value of non-invasive diabetes detectors in the early screening of large-scale diabetes in the Chinese population by extensively carrying out a non-invasive and rapid skin detection project of AGEs in community hospitals.

## Methods

### Study design

Based on the theory that the accumulation of AGEs is closely related to T2DM and vascular complications and supported by the new non-invasive detection technology for diabetes, this study carried out non-invasive and rapid detection of AGEs and other related detection projects in the community. This cross-sectional study aimed to explore the relationship between health risk factors and the diabetes epidemic. The current study was designed and reported according to the Strengthening the Reporting of Observational Studies in Epidemiology (STROBE) checklist.

### Settings

Three community health service centers in Hefei City that signed a medical association agreement with our hospital were selected to screen people aged 30–90 years (excluding those with a clear diagnosis of diabetes and those in the final stage of various diseases) and signed informed consent. Trained investigators used uniformly designed forms to conduct a questionnaire, which recorded the sex, age, height, weight, abdominal circumference, blood pressure, smoking history, disease history, and other information of participants. In addition, fasting blood sugar (FPG), hemoglobin A1c (HbA1C), C-peptide, cholesterol (TC), and triglyceride (TG) levels were determined at 7:30 am. Meanwhile, an oral glucose tolerance test (OGTT) was performed to measure venous plasma glucose and C-peptide levels after glucose loading for 2 h.

### Exposure and outcomes

Doctors and personnel with professional training in designated physical examination institutions had undergone physical checkups based on standard procedures, such as height, weight, AGEs test, fundus examination, blood pressure, and blood and urine samples. Sex, age, height, weight, waist circumference (WC), hip circumference, blood pressure, and other patient characteristics were recorded. Weight was measured, with the participants in underwear, to the nearest 0.1 kg using Soehnle electronic scales, and height was measured in bare feet to the nearest 1 mm using a stadiometer. BMI and waist-to-hip ratio (WHR) were also calculated. We measured systolic blood pressure (SBP) and diastolic blood pressure (DBP) twice in a sitting position after 5 min rest using Hawksley random zero sphygmomanometers. The average of the two readings was considered as the measured blood pressure. Fasting venous blood was collected to determine FPG, 2-h plasma glucose (2hPG), HbA1c, insulin, TC, TG, high-density lipoprotein (HDL-C), creatinine (Cr), urea acid (UA) levels, and C-peptide was tested by OGTT. In addition, other indicators were tested using blood serum. These risk factors are collectively referred to as metabolic indexes.

### Measurement of advanced glycation end products

This study adopted the patented technology of Anhui Yikangda Optoelectronic Technology Co., Ltd. Invention Patent No.: ZL201310173306.2. The excitation light source of the system had a peak wavelength of 370 nm and a half-width of 25 nm. The optical fiber probe can collect diffuse and fluorescence spectra at 350–600 nm. The SAF reflected the AGEs accumulation level in the participants, and the diffuse reflection spectrum was used to correct the influence of individual skin differences on the measurement results. The AGEs fluorescence evaluation index of the skin is the ratio of the integral intensity of the fluorescence spectrum to that of the diffuse reflection spectrum. On the morning of the same day as FBG detection, the AGEs fluorescence spectrum detection system was used to detect the skin fluorescence on the right forearm of the participants (Patent Number: 201320255184.7). During the measurement, obvious blood vessels, scars, and malformed skin areas should be avoided, and the selected test positions should be wiped with alcohol. The fluorescence spectral intensity of the AGEs on the skin of the participants (units: AU) was obtained. Each participant was measured three times, and the average value was used as the final skin AGEs detection result.

### Criteria for risk factors

Hypertension was defined as SBP ≥ 140 mmHg or DBP ≥ 90 mmHg (WHO 1999 criteria) [18] or current use of medications to lower blood pressure. Hyperlipidemia was defined as serum concentrations of TC > 5.69 mmol/L or TG > 1.68 mmol/L or HDL > 1.03 mmol/L or patients that have been treated with medications for lowering lipids. Overweight was defined as BMI > 24 kg/m^2^ and < 28 kg/m^2^. Obesity was defined as BMI ≥ 28 kg/m^2^ based on Chinese standards [19]. The elevated HbA1c level was > 7.0% [20]. Hyperuricemia was defined as serum UA levels ≥ 416 mmol/L (≥ 7.0 mg/dL) in men and ≥ 386 mmol/L (≥ 6.5 mg/dL) in women or if they were treated with allopurinol to lower UA levels [21]. The cut-off age was 65 years [22]. The diagnosis of diabetes complies with FBG ≥ 7.0 mmol/L, and impaired glucose regulation with FPG ≥ 6.1 mmol/L or < 7.0 mmol/L or 2hPG > 7.8 mmol/L or < 11.1 mmol/L [23].

The inclusion criteria were as follows: (1) obtained informed consent from the participants, (2) participants had no history of mental illness, (3) aged 18 years and older and had lived in the survey area for at least 6 months during the 12 months before the survey, and (4) completed the survey on their own.

The exclusion criteria were as follows: (1) participants with a previous history of severe diabetic complications; (2) severe cardiovascular and cerebrovascular diseases; (3) liver and kidney function impairment, infection, or stress, (4) and end-stage population with various diseases.

### Sample size estimation

**Fig. 1:**
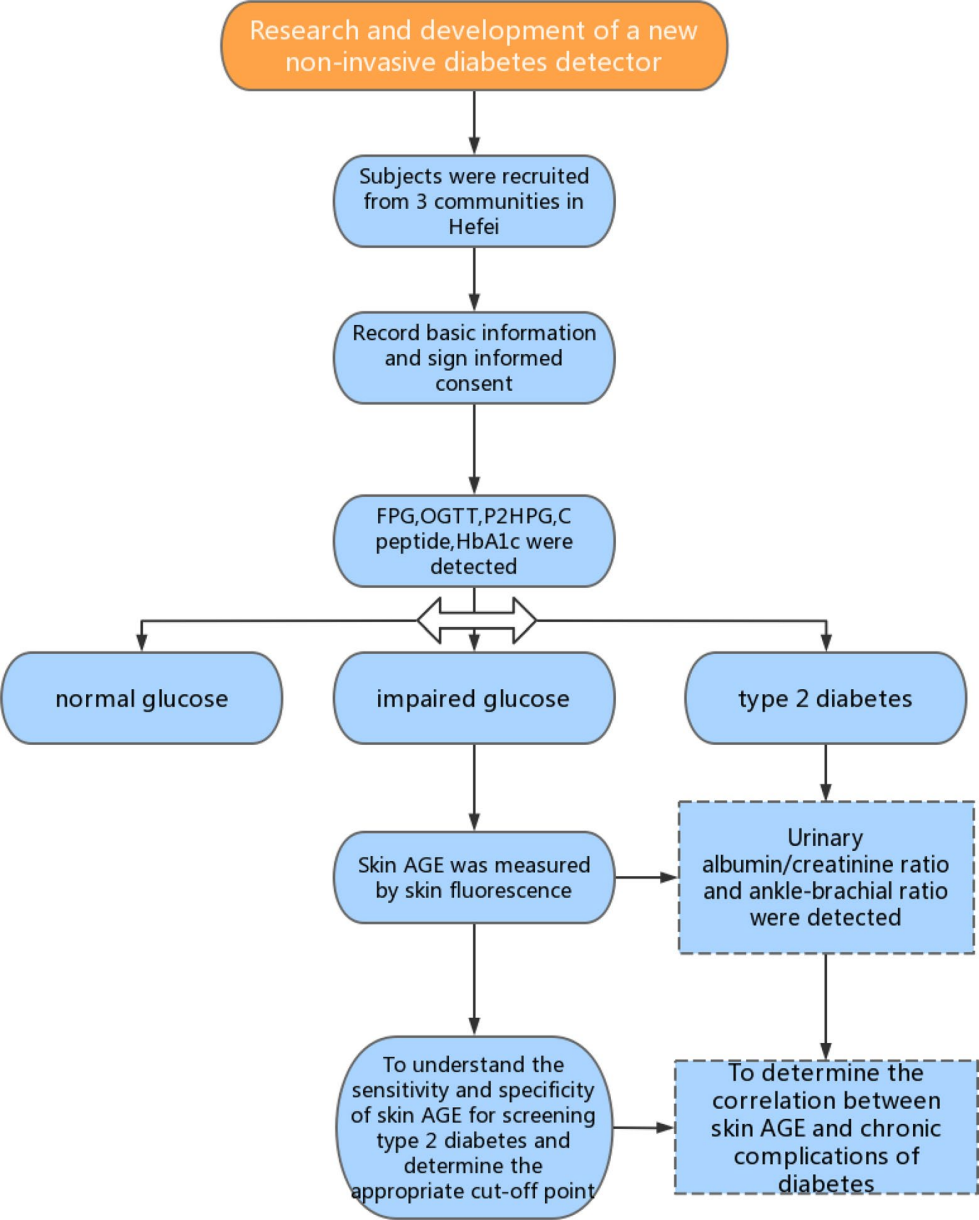
Flow chart of the study participants

Based on previous research, the prevalence of prediabetes was 35.7%, with a relative precision of 15% (*ɛ*), α = 0.05, and Z_1-α/2_ = 1.96. Using the following formula, the minimum sample size was 161. Considering a multicenter design with different ages and communities, analysis and future follow-up are needed. This minimum requirement was used for grade sampling in all regions to ensure that the analysis was performed at multiple stratification levels for each community. Therefore, a total of 700 people were surveyed. A flowchart of the study is shown in Fig. 1.

$$\mathrm{n}=\frac{{(1-\mathrm{p})\mathrm{ Z}}_{1-\mathrm{\alpha }/2}}{{\varepsilon }^{2}p}$$

### Statistics analysis

All data were analyzed using Statistical Packages for the Social Sciences (SPSS) 23.0. Demographic and clinical characteristics of participants were presented as mean ± standard deviation or median with interquartile range (IQR) for skewed data. Multivariate logistic regression analysis was conducted to determine the correlations between different factors and the risk of prediabetes and diabetes after controlling for potential confounding factors.

The statistical analyses were performed in three steps. Step 1 entailed the descriptive statistics pertaining to the health risk factor by the three glucose groups, and the chi-squared test was used to compare categorical variables. In step 2, multilevel logistic regression was used to test the associations between risk factors and the three glucose groups. In step 3, moderation analysis was performed using the PROCESS program of moderation. To test the moderating effect, the relationships for (a), (b), and (c) had to be significant: (a) direct effect of predictor (health risk factors) on the three glucose groups, (b) direct effect of the moderator (BMI) on the three glucose groups, and (c) direct interaction effect (health risk factors × AGEs × BMI) on the three glucose groups. In SPSS PROCESS, the interacting effect is calculated automatically via the software, and it also produces the proportion of variance explained by the moderating effect of BMI (R^2^ increases due to interaction). We adjusted for the effects of sociodemographic correlates (age, sex, DBP, WC, and hip circumference) in the moderation model. I. In addition, we explored whether BMI moderated the relationship between AGE accumulation and the three glucose groups: TG, TC, and HDL. When the 95% confidence interval (CI) did not contain zero, the moderating effect was considered significant [24]. This study was approved by the hospital ethics committee (Ethics Approval No. PJ2019-09-05), and all participants provided written informed consent.

### Sensitivity analysis

In this study, sensitivity analysis was used to test the robustness of the model: (1) Model 1 did not adjust for covariables; Model 2 adjusted for covariates; (2) in this study, we also explored the interactive relationship between AGE, metabolic indexes, and glucose level; and (3) the homeostatic model assessment of insulin resistance (HOMA-IR) index was calculated according to fasting insulin concentration (µU/L) × fasting glucose concentration (mmol/L)/22.5 [25]. The triglyceride glucose (TyG) index was calculated using the natural logarithm formula Ln [TG (mg/dL) × FBG (mg/dL)] / 2 [26]. TG/HDL-C was calculated as TG (mg/dL) divided by HDL-C (mg/dL), and TyG-WC and TyG-BMI were calculated as TyG index × WC and TyG index × BMI, respectively [27]. Visceral adiposity index (VAI): Men: [WC/(39.68 + 1.88 × BMI)] × (TG/1.03) × (1.31/HDL); women: [WC/(36.58 + 1.89 × BMI)] × (TG/0.81) × (1.52/HDL), where both TG and HDL levels are expressed in mmol/L [28]. This study was approved by the ethics committee of the First Affiliated Hospital of Anhui Medical University (Ethics Approval No: PJ2019-09-05), and all participants provided written informed consent.

## Results

### Prevalence characteristics of the three glucose group

A total of 912 patients with a community health physical examination and no history of diabetes were selected, excluding those with missing values > 5%. Finally, 906 samples were included in the study with an effective rate of 99.3%. The prevalence in the normal, impaired glucose tolerance, and DM groups were 79.8%, 10.0%, and 10.2%, respectively. The mean age was 55.34 ± 14.19 years. Older patients (*χ*^2^ = 231.11) were more likely to have a higher prevalence of AGE accumulation. There was no correlation between sex and AGE accumulation (*χ*^2^ = 1.94) (data not shown).

Table 1 presents the results of the risk factor and three glucose groups, which showed that higher AGE accumulation (*χ*^2^ = 37.95), BMI (*χ*^2^ = 12.20), SBP (*χ*^2^ = 8.46), TG (*χ*^2^ = 6.23), and older age (*χ*^2^ = 20.11) were more likely to have a higher prevalence of glucose level; other factors such as HDL (*χ*^2^ = 1.51), UA (*χ*^2^ = 2.69), TC (*χ*^2^ = 2.28), and sex (*χ*^2^ = 5.23) were not correlated with the three glucose groups.

**Table 1:** The prevalence characteristics of three glucose group

	Normal	Impaired glucose tolerance(IGT)	DM	*χ*^2^ value
Age				
< 65	541(82.80%)	63(9.60%)	49(7.50%)	20.11**
≥ 65	173(71.50%)	27(11.20%)	42(17.40%)	
Gender				5.23
Male	378(77.50%)	59(12.10%)	51(10.50%)	
Female	345(82.50%)	32(7.70%)	41(9.80%)	
BMI				12.20**
Normal	402(83.60%)	41(8.50%)	38(7.90%)	
Overweight	257(76.90%)	39(11.70%)	38(11.40%)	
Obesity	64(71.10%)	10(11.10%)	16(17.80%)	
HDL				
Normal	669(80.00%)	85(10.20%)	82(9.80%)	1.51
Abnormal	54(77.10%)	6(8.60%)	10(14.30%)	
UA				2.69
Normal	542(80.70%)	61(9.10%)	69(10.30%)	
Abnormal	181(77.40%)	30(12.80%)	23(9.80%)	
TC				2.28
Normal	624(80.00%)	81(10.40%)	75(9.60%)	
Abnormal	99(78.60%)	10(7.90%)	17(13.50%)	
TG				6.23*
Normal	518(82.00%)	58(9.20%)	56(8.90%)	
Abnormal	205(74.80%)	33(12.00%)	36(13.10%)	
SBP				27.72**
Normal	577(83.40%)	64(9.20%)	51(7.40%)	
Abnormal	143(68.40%)	27(12.90%)	39(18.70%)	
AGE				37.95**
≤ P25	193(84.60%)	23(10.10%)	12(5.30%)	
P25–P50	191(84.90%)	23(10.20%)	11(4.90%)	
P50–P75	177(77.60%)	27(11.80%)	24(10.50%)	
> P75	162(72.00%)	18(8.00%)	45(20.00%)	

*P < 0.05

**P < 0.01

### Multilevel logistic regression between the risk factors and three glucose group

According to multivariate variable analysis, AGE accumulation was divided into ≤ P25, P25–P50, P50–P75, and > P75 using the quartile method. AGE accumulation was positively correlated with the DM group: ≤ P25 [odds ratio (OR) = 0.23, 95% CI 0.12, 0.45], P25–P50 (OR = 0.19, 95% CI 0.09, 0.39), and P50–P75 (OR = 0.50, 95% CI 0.29, 0.85).

After adjusting for sex, age, BMI, and DBP, AGE accumulation was positively correlated with the DM group: ≤ P25 (OR = 0.34, 95% CI 0.15, 0.79) and P25–P50 (OR = 0.29, 95% CI 0.13, 0.64), except for P50–P75 (OR = 0.65, 95% CI 0.36, 1.17). Similarly, after adjusting for sex, age, BMI, DBP, TG, TC, HDL, UA, Cr, and BUN, AGE accumulation was positively correlated with the DM group: ≤ P25 (OR = 0.33, 95% CI 0.14, 0.78) and P25–P50 (OR = 0.30, 95% CI 0.13, 0.66), except for P50–P75 (OR = 0.65, 95% CI 0.36, 1.18). The results are shown in Table 2.

**Table 2:** The interaction effect between AGE accumulation and SBP, HDL, TG among three glucose group

AGE	Model 1			Model 2			Model 3		
	Normal	IGT	DM	Normal	IGT	DM	Normal	IGT	DM
≤ P25	1.0	1.13(0.58,2.18)	0.23(0.12,0.45)**	1.0	1.7(0.76,3.79)	0.34(0.15,0.79)**	1.0	1.74(0.77,3.97)	0.33(0.14,0.78)**
P25–P50	1.0	1.13(0.59,2.20)	0.19(0.09,0.39)**	1.0	1.62(0.77,3.38)	0.29(0.13,0.64)**	1.0	1.69(0.79,3.59)	0.3(0.13,0.66)**
P50–P75	1.0	1.44(0.76,2.75)	0.5(0.29,0.85)**	1.0	1.82(0.92,3.59)	0.65(0.36,1.17)	1.0	1.84(0.92,3.68)	0.65(0.36,1.18)
> P75	1.0	1.0	1.0	1.0	1.0	1.0	1.0	1.0	1.0

Model 1: crude model; Model2: gender, age, BMI, DBP; Model 3: gender, age, BMI, DBP, TG, TC, HDL, UA, Cr, BUN

*P < 0.05

**P < 0.01

### Moderation analysis

Moderation analyses were performed using age, sex, DBP, WC, and hip circumference as control variables. The results are shown in Table 3. First, in Model 1, AGE accumulation significantly predicted the severity of blood glucose (β = 0.071, *P* < 0.05). There was also a dose-dependent trend; TG was not associated with blood glucose (β = − 0.11, *P* > 0.05), and BMI had no correlation (β = − 0.074, *P* > 0.05). In Model 2, after adjusting for age, sex, DBP, WC, and hip circumference, there was no correlation with blood glucose. Secondly, in moderation analysis, BMI moderated blood glucose levels as a result of AGE (β = 0.086, *P* < 0.05). With the increase in BMI level, the higher the AGE, the higher the blood glucose level. However, TG did not moderate blood glucose levels as a result of AGE (β = 0.046, *P* < 0.05). Instead, TG moderated blood glucose levels as a result of BMI (β = 0.33, *P* < 0.05). With the increase in TG level, the higher the BMI, the higher the blood glucose level. Finally, there was a significant three-way interaction among AGE, BMI, and TG for glucose levels in the total sample (β = − 0.16, *P* < 0.01). The results were also significant after adjusting for age, sex, DBP, WC, and hip circumference.

**Table 3:** Model characteristics for the moderation analysis

	Model 1			Model 2		
	*B*	*t* value	*P* value	*B*	*t* value	*P* value
AGE	0.17	1.24	> 0.05	0.19	1.35	> 0.05
BMI	0.61	1.62	> 0.05	0.66	1.69	> 0.05
TC	3.39	1.70	> 0.05	3.93	1.86	> 0.05
Int 1	− 0.01	− 1.51	> 0.05	− 0.01	− 1.54	> 0.05
Int 2	− 0.05	− 1.76	> 0.05	− 0.06	− 1.87	> 0.05
Int 3	− 0.17	− 2.09	** < 0.05**	− 0.18	− 2.14	** < 0.05**
Int 4	0.003	2.17	** < 0.05**	0.003	2.16	** < 0.05**
R^2^						
F						

Model 1: Crude model, model 2: The model was controlled for waist circumference, hip circumference. Independent variables: AGE, dependent variables: FBG

Int 1: AGE × BMI; Int 2: AGE × TG; Int 3: TG × BMI; Int 4: AGE × TG × BMI

In Model 1, AGE significantly predicted the severity of blood glucose (β = 0.095, *P* < 0.01), and there was also a dose-dependent trend (β = 0.42, *P* < 0.05); BMI had no correlation (β = 0.12, *P* > 0.05) with blood glucose. In Model 2, after adjusting for age, sex, DBP, WC, and hip circumference, there was no correlation with blood glucose. Secondly, in moderation analysis, BMI (β = − 0.01, *P* > 0.05) and TG (β = − 0.088, *P* > 0.05) did not moderate blood glucose levels as a result of AGE. SBP did not moderate blood glucose levels as a result of BMI (β = − 0.32, *P* > 0.05). With the increase in SBP level, the higher the BMI, the higher the blood. There was also a significant three-way interaction among AGE, BMI, and SBP for glucose levels in the total sample (β = 0.15, *P* < 0.05). The results were also significant after adjusting for age, sex, DBP, WC, and hip circumference. The results are shown in Table 4.

**Table 4:** Model characteristics for the moderation analysis

	Model 1			Model 2		
	*B*	*t* value	*P* value	*B*	*t* value	*P* value
AGE	0.43	2.49	** < 0.05**	0.41	2.37	** < 0.05**
BMI	1.37	2.79	** < 0.01**	1.29	2.61	** < 0.01**
SBP	0.27	2.77	** < 0.01**	0.25	2.55	** < 0.05**
Int 1	− 0.02	− 2.84	** < 0.01**	− 0.02	− 0.71	** < 0.01**
Int 2	− 0.004	− 2.82	** < 0.01**	− 0.004	− 2.73	** < 0.01**
Int 3	− 0.01	− 3.07	** < 0.01**	− 0.01	− 2.90	** < 0.01**
Int 4	0.0002	3.25	** < 0.01**	0.0002	3.14	** < 0.01**
R^2^	0.09			0.10		
F	13.24			9.33		

Model 1: Crude model, model 2: The model was controlled for gender, age, DBP, hip circumference. Independent variables:AGE, dependent variables: FBG

Int 1: AGE × BMI; Int 2: AGE × SBP; Int 3: SBP × BMI; Int 4: AGE × SBP × BMITable 5 Model characteristics for the moderation analysis

In Model 1, AGE significantly predicted the severity of blood glucose (β = 0.078, *P* < 0.01), and there was also a dose-dependent trend; HDL was not associated with blood glucose (β = − 0.36, *P* > 0.05), and BMI had no correlation (β = 0.027, *P* > 0.05). In Model 2, after adjusting for age, sex, DBP, WC, and hip circumference, there was no correlation with blood glucose. Secondly, in the moderation analysis, BMI (β = 0.04, *P* > 0.05) and HDL (β = 0.17, *P* > 0.05) did not moderate blood glucose levels as a result of AGE. HDL moderated blood glucose levels as a result of BMI (β = − 0.32, *P* > 0.05). With the increase in HDL level, the higher the BMI, the higher the blood glucose level. There was also a significant three-way interaction among AGE, BMI, and HDL for glucose level in the total sample (β = − 0.26, *P* < 0.05). The results were also significant after adjusting for age, sex, DBP, WC, and hip circumference. The results are shown in Table 5.

**Table 5:** Model characteristics for the moderation analysis

	Model 1			Model 2		
	*B*	*t* value	*P* value	*B*	*t* value	*P* value
AGE	0.11	2.20	** < 0.05**	0.11	2.14	** < 0.05**
WC	0.12	2.68	** < 0.01**	0.12	2.54	** < 0.05**
TG	8.20	3.30	** < 0.01**	8.06	3.17	** < 0.05**
Int 1	− 0.001	− 2.29	** < 0.01**	− 0.001	− 2.24	** < 0.01**
Int 2	− 0.12	− 3.43	** < 0.01**	− 0.12	− 3.33	** < 0.01**
Int 3	− 0.11	− 3.84	** < 0.01**	− 0.10	− 3.70	** < 0.01**
Int 4	0.002	4.04	** < 0.01**	0.002	3.91	** < 0.01**
R^2^	0.11			0.13		
F	15.61			11.87		

Model 1: Crude model, model 2: The model was controlled for gender, age, DBP, hip circumference. Independent variables:AGE, dependent variables: FBG

Int 1: AGE × BMI; Int 2: AGE × SBP; Int 3: SBP × BMI; Int 4: AGE × SBP × BMI

### Sensitivity analyses

#### Interaction effect

The interaction between lower AGE accumulation and lower TG was negatively correlated with the DM group (OR = 0.14, 95% CI 0.05, 0.37). Similar results were also found in SBP (OR = 0.07, 95% CI 0.03, 0.18) and HDL (OR = 0.20, 95% CI 0.05, 0.83) levels. After adjusting for sex, age, BMI, and DBP, the interaction between lower AGE accumulation and lower TG were negatively correlated with the DM group (OR = 0.14, 95% CI 0.05, 0.37). The results are shown in Table 6.

**Table 6:** The interaction effect between AGE accumulation and SBP, HDL, TG among three glucose group

Variables	Model 1			Model 2		
	Normal	IGT	DM	Normal	IGT	DM
AGE*TG						
Low AGE* low TG		1.36(0.43,4.31)	0.14(0.05,0.37)**		2.41(0.69,8.40)	0.28(0.09,0.83)**
High AGE* high TG		1.0	1.0		1.0	1.0
AGE*HDL						
Low AGE* low HDL		1.32(0.16,10.68)	0.20(0.05,0.83)**		2.08(0.24,17.88)	0.35(0.08,1.61)
High AGE* high HDL		1.0	1.0		1.0	1.0
AGE*SBP						
Low AGE* low SBP		0.84(0.30,2.41)	0.07(0.03,0.18)**		2.18(0.6,7.92)	0.14(0.05,0.45)**
High AGE* high SBP	1.0	1.0	1.0	1.0	1.0	1.0

Model 1: crude model; Model2: gender, age, BMI, DBP

*P < 0.05

**P < 0.01

#### Correlation between HOMA-IR, TyG index, and TG/HDL-C

HOMA-IR was significantly positively correlated with the TyG index (r = 0.144; *P* < 0.001), and TG/HDL-C (r = 0.462; *P* < 0.001) showed a similar correlation. The TyG index and TG/HDL-C ratio showed a moderately positive correlation (r = 0.462; *P* < 0.001). There was a positive correlation between the TyG index, TC, TG/HDL-C, and glucose levels (β = 4.01, *P* < 0.001). The results are shown in Tables 7, 8, 9, 10.

**Table 7:** Model characteristics for the moderation analysis

	Model 1			Model 2		
	*B*	*t* value	*P* value	*B*	*t* value	*P* value
AGE	0.04	0.82	> 0.05	0.04	0.89	> 0.05
WC	0.05	1.29	> 0.05	0.05	1.29	> 0.05
TG/HDL	8.68	3.52	** < 0.05**	8.77	2.48	** < 0.05**
Int 1	− 0.0004	− 0.78	> 0.05	− 0.001	− 0.85	> 0.05
Int 2	− 0.13	− 2.64	** < 0.05**	− 0.13	− 2.66	** < 0.01**
Int 3	− 0.11	− 2.95	** < 0.01**	− 0.11	− 2.94	** < 0.01**
Int 4	0.001	3.16	** < 0.01**	0.002	3.15	** < 0.01**
R^2^	0.10			0.12		
F	14.35			11.11		

Model 1: Crude model, model 2: The model was controlled for age, gender, DBP, waist circumference, hip circumference. Independent variables: AGE, dependent variables: FBG

Int 1: AGE × WC; Int 2: AGE × HDL; Int 3: HDL × WC; Int 4: AGE × HDL × WC

**Table 8:** Model characteristics for the moderation analysis

	Model 1			Model 2		
	*B*	*t* value	*P* value	*B*	*t* value	*P* value
AGE	0.07	1.22	> 0.05	0.06	1.04	> 0.05
BMI	0.27	1.80	> 0.05	0.25	1.68	> 0.05
TyG	4.07	1.63	> 0.05	3.65	1.41	> 0.05
Int 1	− 0.005	− 2.25	** < 0.05**	− 0.005	− 2.12	** < 0.05**
Int 2	− 0.07	− 1.83	> 0.05	− 0.06	− 1.58	> 0.05
Int 3	− 0.26	− 2.59	** < 0.01**	− 0.26	− 2.42	** < 0.01**
Int 4	0.005	3.17	** < 0.01**	0.005	2.95	** < 0.01**
R^2^	0.32			0.32		
F	59.14			38.47		

Model 1: Crude model, model 2: The model was controlled for age, gender, DBP, waist circumference, hip circumference. Independent variables: AGE, dependent variables: FBG

Int 1: AGE × BMI; Int 2: AGE × TyG; Int 3: BMI × TyG; Int 4: AGE × BMI × TyG

**Table 9:** Model characteristics for the moderation analysis

	Model 1			Model 2		
	*B*	*t* value	*P* value	*B*	*t* value	*P* value
AGE	0.24	2.98	** < 0.01**	0.21	2.59	** < 0.01**
WC	0.21	3.11	** < 0.01**	0.19	2.72	** < 0.01**
TyG	12.37	4.28	** < 0.01**	11.17	2.59	** < 0.01**
Int 1	− 0.003	− 3.54	** < 0.01**	− 0.003	− 3.17	** < 0.01**
Int 2	− 0.18	− 3.06	** < 0.01**	− 0.16	− 2.72	** < 0.01**
Int 3	− 0.16	− 3.34	** < 0.01**	− 0.15	− 3.09	** < 0.01**
Int 4	0.003	3.83	** < 0.01**	0.0024	3.53	** < 0.01**
R^2^	0.31			0.32		
F	58.26			38.30		

Model 1: Crude model, model 2: The model was controlled for age, gender, DBP, waist circumference, hip circumference. Independent variables: AGE, dependent variables: FBG

Int 1: AGE × WC; Int 2: AGE × HDL; Int 3: HDL × WC; Int 4: AGE × HDL × WC

**Table 10:** Model characteristics for the moderation analysis

	Model 1			Model 2		
	*B*	*t* value	*P* value	*B*	*t* value	*P* value
AGE	− 0.17	− 4.97	** < 0.01**	− 0.17	− 4.88	** < 0.01**
VSI	− 3.67	− 4.87	** < 0.01**	− 3.81	− 5.0	** < 0.01**
Gender	− 6.23	− 3.28	** < 0.01**	1.89	− 3.24	** < 0.01**
Int 1	0.06	5.47	** < 0.01**	0.06	5.48	** < 0.01**
Int 2	0.10	3.51	** < 0.01**	0.10	3.56	** < 0.01**
Int 3	1.87	3.48	** < 0.01**	2.0	3.74	** < 0.01**
Int 4	− 0.03	− 3.84	** < 0.01**	− 0.03	− 4.07	** < 0.01**
R^2^	0.08			0.12		
F	10.70			10.98		

Model 1: Crude model, model 2: The model was controlled for age, DBP, waist circumference, hip circumference. Independent variables: AGE, dependent variables: FBG

Int 1: AGE × VSI; Int 2: AGE × gender; Int 3: VSI × gender; Int 4: AGE × VSI × gender

## Discussion

### Research significance

First, the overall prevalence of T2DM and impaired glucose tolerance were 10.2% and 10.0%, respectively, similar to a previous study [1]. Moreover, we also found that AGE accumulation was positively correlated with the DM group, and the high reactivity of fructose (either directly or through its metabolite) may contribute to the formation of AGEs in cells and vascular complications [29]. There was a correlation between AGE, metabolic index, and DM group, which is supported by previous studies that demonstrated methods of preventing diabetes and prediabetes risk by modeling risk factors [30]. Second, older age is associated with AGE accumulation, while AGEs accumulate in the body during aging; therefore, it is necessary to provide preventive measures for older patients with more complications [31]. Older age and obesity are also associated with high glucose levels [32, 33]. Finally, in moderation analysis, BMI moderated the correlation between AGE and glucose levels, and SBP, TG, and HDL also moderated this relationship. Preventive interventions for diabetes also exist, prompting recent assessments of risk scores (including those studied here) to predict diabetes [30]. Moreover, we verified our robustness based on sensitivity analysis results.

Similarly, in a study of 172 healthy individuals, age and sex differences were found to be associated with AGE. Serum AGEs have been reported in the range of 8.5 ± 0.9 units/mL in men aged < 45 years, 9.9 ± 1.5 units/mL in men aged > 60 years, 7.9 ± 0.7 units/mL in women aged < 45 years, and 10.7 ± 1.1 units/mL in women aged > 60 years [34]. Generally, AGE levels are stringently related to blood glucose and other metabolic indexes. There seems to be an interactive association between AGEs and diabetic micro- and macrovascular complications related to TG, TC, and SBP levels on glucose levels. The explanation for the increased concentration of AGEs in patients with DM is oxidative stress and inflammatory response [35]. Many studies have demonstrated an association between elevated AGE levels and cardiovascular disease in patients with DM, and SAF has also been associated with macrovascular complications in patients with T2DM [36]. Moreover, the strength of AGEs was that AGEs had been implicated in the long-term nature of metabolic memory, and their assessment takes into account cumulative glycemic exposure and glycemic variability, thus, overcoming the limitations of HbA1c as a biomarker for diabetic outcomes [37]. However, the association of SAF with diabetes and its complications does not necessarily mean that this is a causal relationship or that interventions aimed at decreasing SAF will reduce chronic organ complications. Therefore, it is possible that AGEs not only predict diabetes and its complications but also are a cause of diabetes and its complications. Therefore, intervention trials with agents that decrease AGE accumulation are required to investigate this.

### Correlations between the metabolic index, BMI, AGE accumulation, and three glucose group

Several studies have focused on the correlation between cardiovascular diseases and AGEs. For example, various skin collagen-linked AGEs in patients with type 1 diabetes were studied and found to be associated with the “metabolic memory” phenomenon reported in the Epidemiology of Diabetes Interventions and Complications (EDIC) study [38]. In another study, HbA1c, plasma Cr, albumin-creatinine ratio, HDL-C, age, sex, smoking, and diabetes duration were all associated with skin AGEs, again showing that this pathway may be especially relevant in certain risk groups [39]. In this study, non-invasive skin AGEs levels were detected in community populations to identify possible influencing factors. The first was the detection of biochemical indicators, including SBP, TC, TG, HDL, BMI, HbA1c, FBG, and AGE. Normally, the generation of AGEs proceeds through a slow glycosylation stage, and hyperglycemia, hyperlipidemia, or oxidative stress can accelerate the accumulation of AGEs in the body [40]. Oxidative stress, caused by the overproduction of reactive oxygen species (ROS), which inhibit glyoxalase-1 (Glo-1) activity by reducing its co-factor, plays an important role in the activation of other pathogenic pathways involved in diabetic complications. In a previous study, Fujimoto demonstrated that a key precursor of AGEs is the dicarbonyl metabolite MG, which is metabolized by Glo1 of the cytoplasmic glyoxalase system [41].

In this study, multivariate logistic regression analysis showed that AGEs were associated with both diabetes and prediabetes risks. Therefore, it could be speculated that elevated levels of AGEs in the skin could be a warning sign of diabetes. High serum HbA1c can accelerate the formation of AGEs and hydroxymethyllysine. Even if HbA1c is well controlled, high levels of AGEs can increase the risk of diabetic microvascular complications and form the "metabolic memory" effect of hyperglycemia [42]. Patients with type 1 diabetes were followed up for 7 years, and AGEs fluorescence spectrum detection of the skin could predict the occurrence of major adverse cardiovascular events in patients with type 1 diabetes. The higher the intensity of AGEs fluorescence in the skin, the higher the probability of myocardial infarction, stroke, lipid metabolism index, lower limb paraplegia, or revascularization surgery in patients with type 1 diabetes [43]. In addition, a previous study proposed that AGEs are associated with the pathophysiology of obesity [44].

### Moderating role of BMI between metabolic index and AGE accumulation among the three glucose groups

Another study has reported that adults with obesity (average BMI 33.2 kg/m^2^) exhibited significantly higher circulating AGEs compared with overweight participants (average BMI 26.3 kg/m^2^) [45]. Serum AGEs were found to correlate with TG levels, and those with the highest AGEs levels had the most adverse lipid profiles [46]. Another study demonstrated that AGE is also associated with diabetes, its complications, and obesity [47]. Elevated AGEs levels have been associated with both systolic and diastolic dysfunction in patients with DM [48], and our results were similar to these results. A possible explanation for this is that AGEs play an important role in oxidative stress-induced apoptosis of capillary pericytes [49]. Another possible explanation for this finding is that AGEs can promote intraocular vascular proliferation and pro-inflammatory responses, mainly through the production of vascular endothelial growth factor (VEGF) and pro-inflammatory cytokines such as interleukins and tumor necrosis factors [47, 50–53]. Therefore, from the perspective of the interaction of risk factors, we explored the potential possibility that AGE leads to elevated blood glucose levels. In our study, we found an interaction effect between AGE and metabolic indexes. A previous study demonstrated that AGE-1 levels were higher in patients with prediabetes/diabetes than that in controls, and its elevation was associated with metabolic syndrome (MetS), obesity, hyperlipidemia, and non-alcoholic fatty liver disease (NAFLD) [54]. Other studies have used the TyG index as a surrogate to identify insulin resistance (IR) [55]. According to the results, the above results further provided a theoretical basis for the study of the moderation analyses’ role in the significance of health risk factors, BMI, AGE, and glucose level.

Our research suggests that participants, especially those with higher AGE accumulation, obesity, and higher health risk factors, seem vulnerable to high glucose levels. From a developmental disease perspective, AGE accumulation and obesity interact with health risk factors, thereby influencing the development of glucose levels. As a moderating variable, BMI has specific effects on the association between AGE accumulation and glucose level, and the quality of health risk factors moderates the relationship between AGE, BMI, and glucose level. In other words, there is a three-way interaction between AGE, health risk factors, BMI, and glucose levels. It is well known that AGEs, HbA1c, and obesity are all associated with glucose levels, and glucose levels may play a more prominent role in the health of individuals with obesity; therefore, obesity may be one of the health risk factors pathophysiological mechanisms leading to increase glucose level in AGE accumulation [47, 56]. This is also reflected in the side, clinical management of diabetes mainly focuses on blood glucose control, blood pressure control, lipid-lowering, lifestyle management, and physically active days [13, 17]. If blood glucose is a major factor in the occurrence of these complications, then by normalizing blood glucose levels [57], diabetes can be cured, and complications can be prevented by 100%. This is one of the reasons why this study explored these risk factors because there may be interactions between them.

In addition, we added health risk factors such as HDL, TG, and SBP according to the diathesis-stress model; higher HDL, TG, and SBP levels were more vulnerable to the higher effects of glucose levels in a higher BMI (obesity), consequently leading to worse outcomes for glucose levels. Although components demonstrating this inverse relationship included BMI, TG, HbA1c, and IR, which were inversely linked with sRAGE (receptor for AGEs) in participants with and without diabetes [58], this also verifies that the indicators selected in this study are reasonable. Gugliucci et al. reported that 90% of the fructose consumed is metabolized by the liver in the first stage, during which it stimulates de novo fat production to drive the synthesis in the liver and fructose-mediated increased methylglyoxal flux (lipogenesis, fatty liver, and IR) leads to hepatic AMP-activated protein kinase inactivation [29]. Therefore, it is necessary to explore the correlation between AGEs, TG, and glucose levels. A previous study found that BMI was significantly associated with SBP and glucose level [59], and Khamseh et al. also demonstrated that the TyG index is strongly related to obesity [55], which provides a theoretical basis for this study to explore the relationship between BMI, health risk factors, and blood glucose levels. Another reason is that AGEs increase with increasing BMI, whereas the link is the opposite for sRAGE. sRAGE may reflect tissue RAGE expression, and that sRAGE may increase along with AGE to mount a counter-defense [60]. These observations suggest that although exogenous AGEs may play a key role in the pathogenesis of these disorders, hyperglycemia and hyperlipidemia resulting from established pathology likely further fuel the AGE-RAGE system, resulting in severe complications [44]. The practical implications of these results suggest that we should focus on the interaction of many factors, not just one.

### Theoretical and clinical significance of the three glucose group

The fluorescence intensity of AGEs in the skin can reflect the longer time of blood glucose control, which may be one of the indicators to predict the chronic complications and mortality of diabetes [44]. Previous studies have shown that the interaction between AGEs and RAGE causes oxidative stress, inflammation, and fibrosis, which can lead to endothelial dysfunction, atherosclerosis, vascular stiffness, progressive changes in renal structure, and impaired renal function [35, 61, 62]. Therefore, non-invasive skin detection of AGEs is of great significance for the prevention and monitoring of diabetes and its complications. Venous blood FPG, OGTT, and HbA1c are important indicators for the screening and diagnosis of diabetes. However, there are some problems, such as invasiveness, high cost, and a long time to transport samples [63, 64]. Nevertheless, a large number of foreign studies have reported that non-invasive devices that detect the fluorescence of skin AGEs can be used for diabetes screening, and AGE is relatively highly sensitive for diabetes diagnosis, with strong operability and extensibility [65–67]. Here, we propose that AGEs are important members of the common soil that affect vascular and metabolic diseases such as diabetes.

## Limitation and strength

The strength of the study includes that a large number of foreign studies reported that SAF could be used for diabetes screening, and the sensitivity of the diagnosis of diabetes is higher than FPG or HbA1c, which gives us a good theoretical basis. In addition, this study is a multicenter and multilevel design and has a high number of participants.

This study had several limitations. The author did not investigate the participants’ family history of diabetes, or dietary habits, which may have affected the results of the study [68]. In addition, Noordzij reported that SAF detection might have some limitations, such as skin pigmentation, use of cream and sunscreen, extreme congestion, and vasoconstriction, which may affect the measurement results [69]. As this was a cross-sectional study, it was difficult to observe the causal association between health risk factors, BMI, AGE, and glucose levels. This study only investigated the results of three community hospitals, and it is not clear how representative this sample may be. The follow-up survey will be carried out among samples from different regions and cultures across the country.

## Conclusion

The present study builds upon the extant literature documenting the possible association between AGE accumulation, BMI, and health risk factors in the context of Chinese culture. In devising interventions for the primary prevention of AGE accumulation among participants, especially among older participants, health risk factors should be considered. Reducing the incidence of health risk factors can significantly reduce BMI. In addition, lower BMI may prevent or reduce AGE accumulation, particularly by targeting older participants. This study also suggests that Chinese participants could use weight control therapy as an adjunct technique for modifying BMI in the treatment of adolescents with higher health risk factors. Moreover, as a moderating variable, BMI varies by sex in the relationship between HFRs and AGE accumulation. The findings highlight the need to focus on normal BMI and employ sex-specific methods to cope with AGE accumulation in adolescents with a history of higher health risk factors. Considering AGEs predict not only complications but also are a cause of complications, we should take care of the importance of AGEs [41].

In general, non-invasive skin detection of AGEs has a certain application value for the assessment of diabetes risk. However, much is yet to be conducted in AGEs research. More studies are needed to confirm this non-invasive technique as a new diabetes screening tool to improve the diagnosis rate of diabetes. A previous study compared plasma, serum, and skin samples and found interesting results that all studies measuring AGEs in skin biopsies found positive results [70]. More recently, a completely non-invasive method of measuring SAF has become available, reflecting some of the AGEs, which will potentially be used more frequently over the next years instead of continuing to use blood plasma, blood cells, or SAF. Clinically most affected tissues such as the kidney, nerves, or retina might be much more promising tissues to be studied. To further evaluate the links between AGE metabolism and diabetic complications, not only epidemiologic studies present, but also interventional studies are interesting and might provide important insights into new pathophysiological links. To this end, significant efforts have been made using experimental models that generally agree with the results of research in humans and highlight the potential direct effect of AGEs on the pathogenesis of obesity, IR, and diabetes. We hope this study ignites further interest in this important and promising line of research, which is an active area of investigation in our laboratories.

## Data Availability

The data used and analyzed during the study are available from the corresponding author on reasonable request.
